# Editorial: Cannabinoid interactions with ion channels, receptors, and the bio-membrane

**DOI:** 10.3389/fphys.2023.1211230

**Published:** 2023-05-09

**Authors:** Mohammad-Reza Ghovanloo, Jonathon C. Arnold, Peter C. Ruben

**Affiliations:** ^1^ Department of Neurology, Yale University School of Medicine, New Haven, CT, United States; ^2^ Center for Neuroscience and Regeneration Research, Yale University, New Haven, CT, United States; ^3^ The Lambert Initiative for Cannabinoid Therapeutics, Brain and Mind Centre, The University of Sydney, Sydney, NSW, Australia; ^4^ Discipline of Pharmacology, Sydney Pharmacy School, Faculty of Medicine and Health, The University of Sydney, Sydney, NSW, Australia; ^5^ Department of Biomedical Physiology and Kinesiology, Simon Fraser University, Burnaby, BC, Canada

**Keywords:** cannabinoid, endocannabinnoid, phytocannabinoid, ion channel, receptor, biomembrane, voltage-gated sodium and calcium channels, CB and CB 1 2

## Background

Cannabinoids are a class of natural compounds that are found in the endocannabinoid system and the cannabis plant. Synthetic cannabinoids have also emerged, often discussed as new psychoactive substances (NPS), however, these cannabinoid analogues are additionally used as tool compounds and as potential novel therapeutic agents.

**FIGURE 1:**
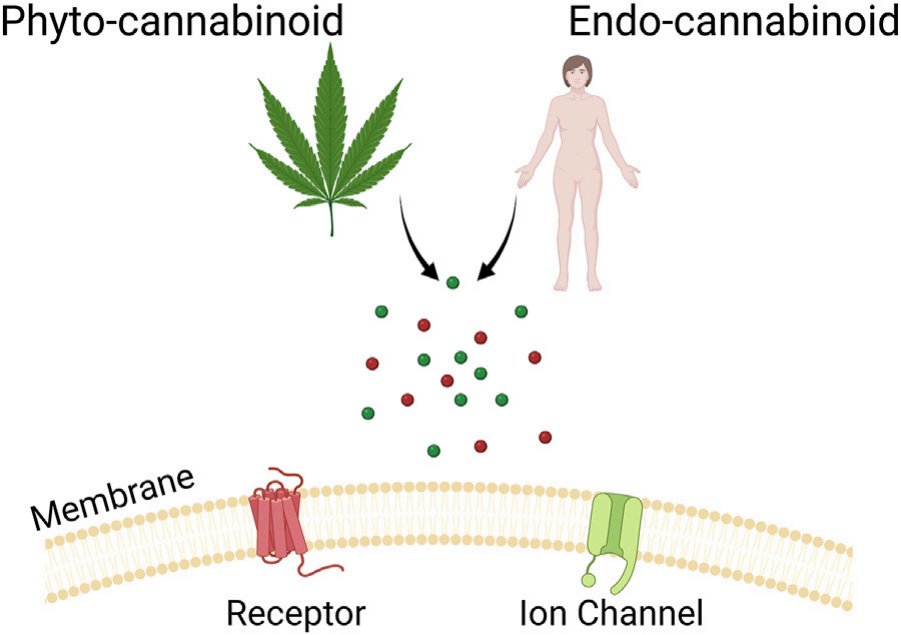
Shows a cartoon representation of the theme of this Research Topic.

The interest in cannabinoid science has grown steadily in recent years, particularly with the legalisation of medicinal cannabis in many jurisdictions around the world. It has become abundantly clear that cannabinoids interact with a host of different receptors and proteins, both within the endocannabinoid system and outside of it (Figure 1) (Pertwee, 2008; Billakota et al., 2019; Ghovanloo and Ruben, 2021). The interactions between these compounds and various molecular targets affect and alleviate a wide variety of physiological processes underlying pain, mood changes, and seizure disorders. Indeed, with the identification of treatment-resistant disorders, and growing public health problems such as the opioid crisis (Krausz et al., 2021), the need to investigate cannabinoids across various disciplines is increasingly urgent; they have already revealed some surprising potential to treat otherwise intractable health problems.

Cannabidiol (CBD) is among the most promising cannabinoids and has shown clinically proven efficacy against epileptic disorders (Devinsky et al., 2017; Devinsky et al., 2018). The intrigue surrounding this compound arises from anecdotal reports of its efficacy against many other disorders and is likely related to the large number of its pharmacological targets. In contrast to ∆^9^-tetrahydrocannabinol (THC), which has low nanomolar affinity and partial agonist effects on endocannabinoid (CB) receptors, CBD has limited binding at CB receptors and complex effects with inverse agonist/antagonist and negative allosteric modulatory effects at CB receptors being described (Thomas et al., 2007; Laprairie et al., 2015). Thus, its mechanism of efficacy, especially against seizure-related disorders, is mainly attributed to targets independent of CB receptors. This concept is at the core of why CBD, as well as other cannabinoids, are increasingly studied against different molecular targets ranging from ion channels to receptors, and even the bio-membrane itself (Wade et al., 2004; Ross et al., 2008; De Petrocellis et al., 2012; Ghovanloo et al., 2018; 2021; 2022c; Harding et al., 2018; Fouda et al., 2020; Orvos et al., 2020; Sait et al., 2020; Zhang and Bean, 2021; Zhang et al., 2022). Because each of these targets has an important role in many physiological processes and disease mechanisms, cannabinoids have therapeutic potential for disorders that originate throughout the body including, but perhaps not limited to, excitable tissues: muscles and nerves (Devane et al., 1988; Elsohly, 2007; Ghovanloo and Ruben, 2021). Indeed, looking beyond CBD and THC, other “minor” plant cannabinoids have been more recently explored for their medicinal potential with preclinical research showing a diversity of potential therapeutic applications including anti-inflammatory, anti-cancer, anti-seizure, anxiolytic, and anti-emetic activities (Anderson et al., 2019; 2021b; 2021a; Assareh et al., 2020; Rock et al., 2021; Colvin et al., 2022; Lowin et al., 2023). However, the modes of action of these cannabis constituents remain to be determined.

Our goal in this Research Topic was to disseminate up-to-date knowledge of cannabinoid science pertaining to activity at different targets, with implications for physiology and the treatment of medical conditions.

## Articles published in Research Topic

A total of five papers appeared in our Research Topic, including both literature reviews and original research articles.

The first paper published this Research Topic by Oz et al. provides a literature review on the effects of cannabinoids on ligand-gated ion channels. This review paper focuses on how various cannabinoids, phytocannabinoids, endocannabinoids, and synthetic cannabinoids, modulate the activity of nicotinic, 5-HT_3_, glycine, and GABA_A_ receptors (Oz et al.).

In the second paper, Schmiedhofer et al. contribute a systematic review on the interactions of cannabinoids with Cys-loop receptors. This paper begins with an overview of cannabinoids followed by an in-depth description of interactions with Cys-loops, which have important molecular consequences. The paper summarizes pharmacological and medicinal implications of cannabinoid/Cys-loop interactions (Schmiedhofer et al.).

The third paper by Ghovanloo et al. is a mini-review on the interactions of two important non-psychoactive compounds, cannabigerol (CBG) and CBD, with voltage-gated sodium (Nav) channels. This paper starts with an overview of CBD’s mechanism of action on Nav channels, and then describes how CBG may have therapeutic potential to treat neuropathic pain, in part, via activity on Nav channels (Ghovanloo et al.; Ghovanloo et al., 2022b).

The fourth paper by Milligan et al. is an original research article that builds on the idea that Nav channels are critical to cannabinoid activity. This paper investigates the inhibitory effects of several lesser studied cannabinoids on Nav channels, including cannabigerolic acid (CBGA), cannabidivarinic acid (CBDVA), cannabichromenic acid (CBCA), and cannabichromene (CBC). These compounds were recently identified as having anti-seizure activity in preclinical epilepsy models, however their modes of action are unclear (Anderson et al., 2019; Anderson et al., 2021a; Anderson et al., 2021b). This paper highlights the importance of gaining a better understanding of the molecular actions of all cannabinoids on not only Nav channels, but other important signalling proteins (Milligan et al.).

Finally, in the fifth paper, an intriguing original research article by Harman et al. provides insights into how MEPIRAPIM-derived synthetic cannabinoids inhibit T-type calcium (Cav3) channels with divergent effects in seizure models. This paper provides a novel avenue for the development of future anti-seizure therapeutics through targeting T-type channels using a synthetic cannabinoid scaffold without CB receptor activity (Harman et al.).

In summary, this Research Topic presents a series of papers focused on various therapeutic and molecular investigations of cannabinoids. Despite much research in this area, there are many questions that remain unanswered, including the synergistic effects of cannabinoids. We hope that this Research Topic inspires an even greater interest in cannabinoid science.
